# The wandering mimic: Meckel’s diverticulum with enterolith simulating an intraperitoneal hydatid cyst

**DOI:** 10.1016/j.radcr.2026.06.032

**Published:** 2026-07-07

**Authors:** Fahad Alhamad, Mohammed Almuhanna, Reem Alanazi, Ahmed Aldraihem, Aljoharah Aljabr

**Affiliations:** Pediatric Radiology Department, King Fahad Medical City, Riyadh, Saudi Arabia

**Keywords:** Meckel's diverticulum, Enterolith formation, Hydatid daughter cysts

## Abstract

Meckel’s diverticulum (MD) is the most common congenital anomaly of the gastrointestinal tract and is often asymptomatic. Unusual complications such as enterolith formation or foreign body impaction are rare, particularly in pediatric patients, and may produce atypical imaging findings that mimic other intra-abdominal pathologies. We report a case of a previously healthy 4-year-old boy who presented with intermittent colicky abdominal pain and non-bilious vomiting. Contrast-enhanced CT demonstrated a cystic lesion in the left paracolic gutter containing multiple internal rounded low-attenuation structures, initially suspicious for hydatid daughter cysts. Follow-up MRI revealed interval migration of the lesion to the right lower quadrant with peripheral rim enhancement and internal floating components, raising suspicion for a mobile bowel-related process. The patient underwent surgical exploration, which confirmed an inflamed Meckel’s diverticulum arising from the ileum. Histopathology demonstrated an intraluminal foreign body (watermelon seed) with surrounding enterolith formation, corresponding to the presumed daughter cysts on imaging. Complicated Meckel’s diverticulum may closely mimic hydatid disease on cross-sectional imaging, particularly when enteroliths and inflammatory debris simulate daughter cysts. Recognition of lesion mobility and careful clinicoradiologic correlation are critical to avoid misdiagnosis and unnecessary antiparasitic therapy. This case highlights an important diagnostic pitfall in pediatric abdominal imaging.

## Introduction

Meckel’s diverticulum (MD) is the most common congenital anomaly of the gastrointestinal tract, resulting from incomplete obliteration of the vitelline duct and occurring in approximately 2% of the population [1]. Although most cases remain asymptomatic, complications such as gastrointestinal bleeding, obstruction, diverticulitis, or perforation may occur [1,2]. Rarely, foreign body impaction or enterolith formation within MD can produce atypical imaging findings and create significant diagnostic challenges, particularly in pediatric patients [3].

In regions where echinococcosis is endemic, cystic intra-abdominal lesions demonstrating internal vesicles (“daughter cysts”) or floating membranes (“water lily sign”) are frequently interpreted as hydatid disease [4]. However, several non-parasitic conditions including complicated Meckel’s diverticulum may closely mimic hydatid cysts on cross-sectional imaging, representing an important diagnostic pitfall [5].

We report a pediatric case of a mobile (wandering) Meckel’s diverticulum with enterolith formation that radiologically simulated hydatid disease.

## Case presentation

A previously healthy 4-year-old boy presented to the emergency department with intermittent abdominal pain and episodic non-bilious vomiting of several weeks’ duration. The pain was colicky, recurrent, and poorly localized. There was no history of fever, weight loss, trauma, prior abdominal surgery, or exposure to livestock or unpasteurized dairy products.

On examination, the patient was hemodynamically stable. The abdomen was mildly distended but soft, without guarding or rebound tenderness. No palpable mass was identified.

Laboratory investigations were largely unremarkable. The white blood cell count was within normal limits without leukocytosis. Mild anemia was noted on subsequent testing. Platelet count, erythrocyte sedimentation rate, and serum electrolytes were within normal limits. Tumor markers (AFP and β-hCG) were normal. Echinococcus serology was negative.

### CT findings

Contrast-enhanced CT of the abdomen demonstrated a left paracolic gutter fluid collection with multiple well defined internal components which could represent daughter cysts.

Additional findings included reactive mesenteric lymph nodes (largest approximately 1 × 0.8 cm), displacement of adjacent bowel loops without evidence of obstruction, and mild surrounding inflammatory changes. No definite communication with adjacent bowel loops was identified on CT. Based on these imaging features, hydatid disease was initially suspected. (Fig. 1, Fig. 2).

**Fig. 1:**
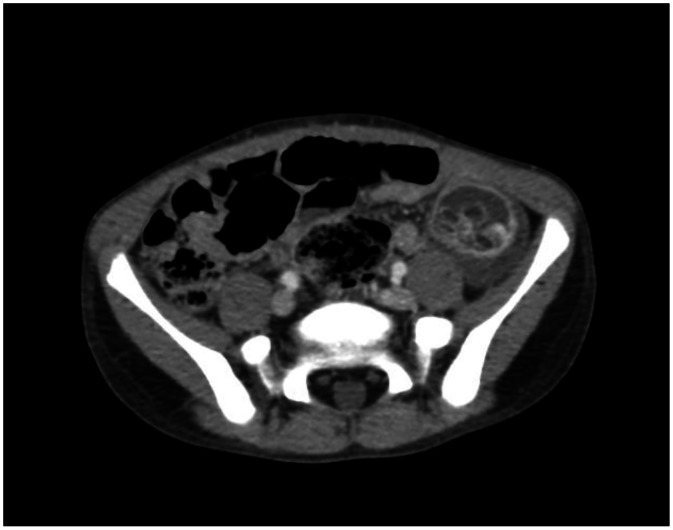
Axial contrast-enhanced CT of the abdomen demonstrates a well-defined cystic lesion in the left paracolic gutter containing multiple internal low-attenuation rounded structures producing a characteristic floating appearance, initially suspicious for hydatid daughter cysts.

**Fig. 2:**
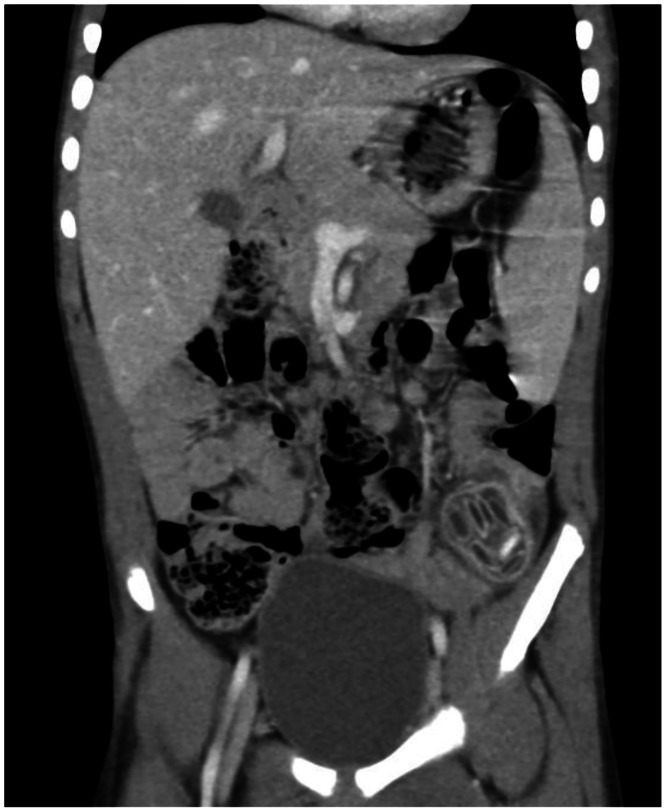
Coronal contrast-enhanced CT image shows the same intraperitoneal cystic lesion with internal clustered vesicle-like components and mild surrounding inflammatory changes, without definite bowel communication.

## MRI findings

Follow-up MRI demonstrated interval migration of the lesion from the left paracolic gutter to the right lower quadrant, indicating lesion mobility. The lesion showed heterogeneous signal on both T1- and T2-weighted images with multiple internal floating rounded components. Focal internal calcification was present.

Post-contrast images demonstrated peripheral rim enhancement with adjacent mesenteric stranding and persistent mild intraperitoneal free fluid. Diffusion-weighted imaging showed no definite restricted diffusion.

The interval positional change raised strong suspicion for bowel-related pathology rather than a fixed peritoneal cyst (Fig. 3, Fig. 4, Fig. 5, Fig. 6, Fig. 7).

**Fig. 3:**
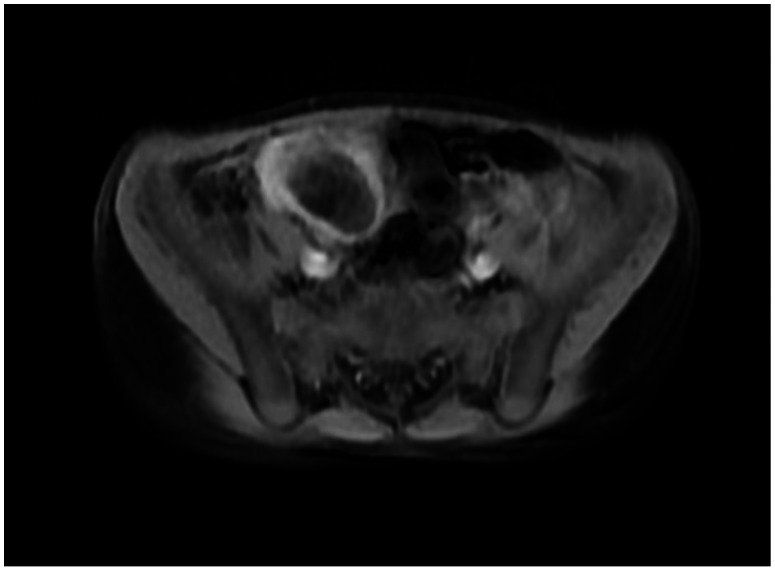
Axial post-contrast T1-weighted MRI shows peripheral rim enhancement of the cystic lesion with adjacent mesenteric stranding, consistent with inflammatory etiology.

**Fig. 4:**
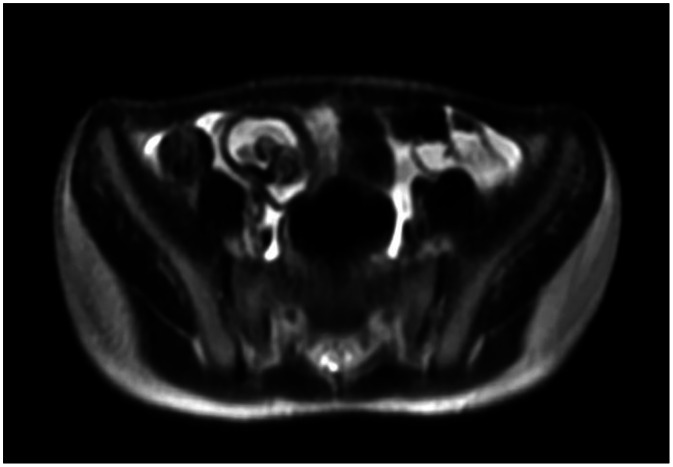
Axial T2-weighted MRI again demonstrates the lesion with internal floating structures and surrounding mild inflammatory changes.

**Fig. 5:**
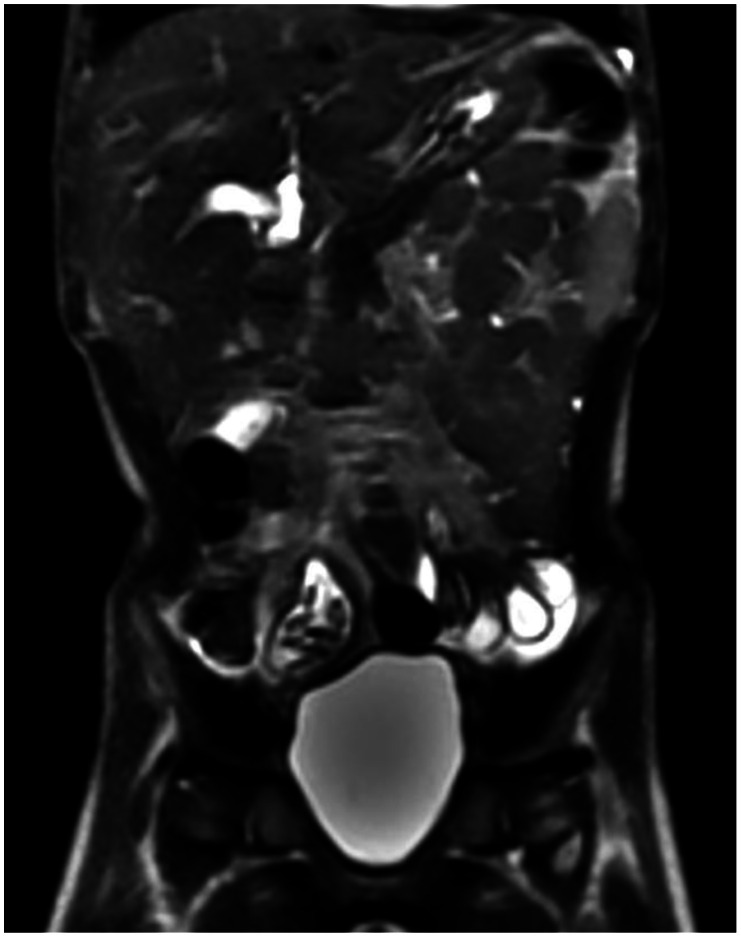
Coronal T2-weighted MRI confirms interval migration of the lesion to the right lower quadrant, supporting a mobile bowel-related process rather than a fixed peritoneal cyst.

**Fig. 6:**
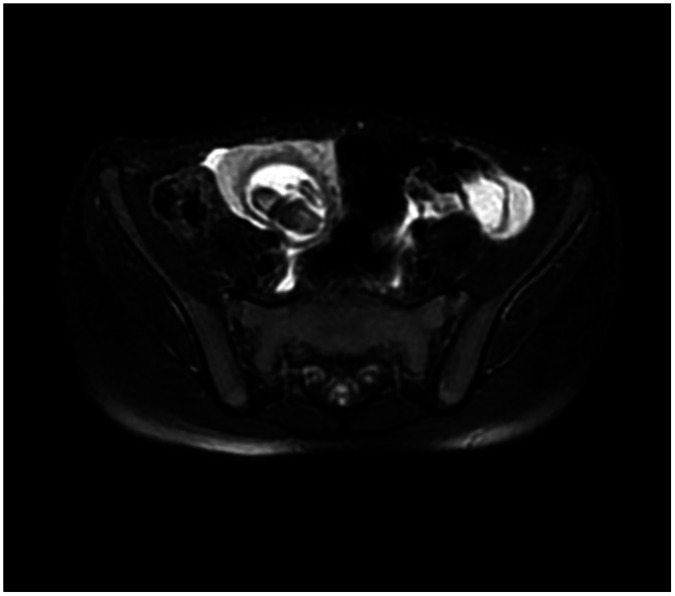
Axial T2-weighted fat-suppressed MRI demonstrates the cystic lesion with heterogeneous high T2 signal and multiple internal rounded floating components within the right lower abdomen.

**Fig. 7:**
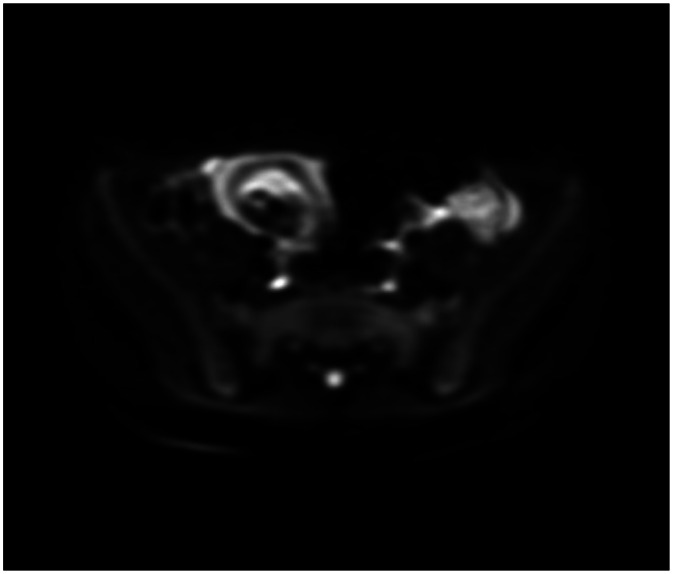
Axial diffusion-weighted imaging (DWI) shows no definite restricted diffusion within the lesion, arguing against a highly cellular abscess.

### Surgical and pathologic findings

The patient underwent surgical exploration. An inflamed Meckel’s diverticulum arising from the ileum was identified and resected.

Histopathologic examination demonstrated:

• Intraluminal foreign body (watermelon seed).
• Enterolith formation surrounding the foreign body.

The presumed daughter cysts on imaging corresponded to inflammatory debris and enterolith fragments within the fluid-filled diverticulum.

## Discussion

This case highlights two important diagnostic pitfalls in pediatric abdominal imaging: the wandering cystic mass and hydatid disease mimicry. Awareness of these features is essential to avoid misdiagnosis and unnecessary treatment.

### Wandering behavior

The interval positional change of the lesion between CT and MRI represents a key diagnostic clue. Meckel’s diverticulum is typically located within approximately 100 cm of the ileocecal valve but may demonstrate considerable mobility when associated with freely mobile small bowel loops or a relatively long mesenteric attachment [1,2].

Recent imaging studies emphasize that apparent migration of an intra-abdominal cystic lesion should raise suspicion for a bowel-related process rather than a fixed peritoneal or mesenteric cyst [5,6].

In the present case, the observed “wandering” behavior reflected mobility of the ileal segment containing the inflamed diverticulum, which helped narrow the differential diagnosis.

### Hydatid mimicry

The clustered internal rounded structures observed on CT closely simulated hydatid daughter cysts, a finding traditionally considered highly suggestive of echinococcosis.⁴ However, several non-parasitic entities—including complicated Meckel’s diverticulum—may demonstrate similar imaging appearances [5].

Enterolith formation within Meckel’s diverticulum is uncommon in pediatric patients but may occur in the setting of luminal stasis and chronic inflammation [3]. In the present case, the retained watermelon seed likely acted as a nidus for enterolith formation, producing the vesicle-like internal architecture that mimicked hydatid daughter cysts.

### Role of multimodality imaging

Multimodality imaging played a crucial role in this case. While CT initially suggested hydatid disease because of the vesicular internal pattern, MRI demonstrated interval lesion migration and peripheral inflammatory enhancement.

Integration of imaging findings with clinical and serologic data—such as negative echinococcal serology and absence of hepatic or pulmonary cysts—was essential for narrowing the diagnosis.

### Clinical implications

Recognition of this diagnostic pitfall is important, particularly in regions where hydatid disease is endemic. Misinterpretation may lead to unnecessary antiparasitic therapy or delays in definitive surgical management. Awareness that complicated Meckel’s diverticulum can present as a mobile cystic lesion with daughter cyst–like internal structures may improve diagnostic accuracy.

## Conclusion

Complicated Meckel’s diverticulum may rarely present as a mobile intraperitoneal cystic lesion and closely mimic hydatid disease on cross-sectional imaging. In this case, intraluminal enterolith formation around an ingested foreign body produced multiple internal rounded structures that simulated hydatid daughter cysts on CT. Interval lesion migration on follow-up MRI, together with peripheral inflammatory changes, provided an important diagnostic clue suggesting a bowel-related inflammatory process rather than a true parasitic cyst. Recognition of this potential imaging pitfall and careful clinicradiologic correlation are essential to avoid misdiagnosis and unnecessary antiparasitic therapy, while facilitating timely surgical management. This case highlights the importance of considering complicated Meckel’s diverticulum in the differential diagnosis of mobile cystic intra-abdominal lesions in pediatric patients.

## Patient consent

Written informed consent for publication was obtained from the patient.
